# What really happens when a dev vibes with the code? An empirical study on LLM behavioral divergence in response to expressive code comments

**DOI:** 10.3389/frai.2026.1784973

**Published:** 2026-08-11

**Authors:** Angela N. Johnson

**Affiliations:** 1 College of Professional Studies, Northeastern University, Boston, MA, United States; 2 Avania, Boston, MA, United States; 3 The Real Cat Labs, Boston, MA, United States

**Keywords:** agentic code, code comment, human–AI collaboration, large language models, software development, vibe coding

## Abstract

**Introduction:**

We investigate how expressive inline code comments written in various developer styles, functional to progressively poetic, philosophical, and misleading, affect large language model (LLM) behavior during code optimization.

**Methods:**

In this pilot study, we used a controlledmerge sort implementation across five stylistic variants and evaluated GPT-5 and Claude Opus 4.1 under standardized console prompts, isolating the effect of embedded comment semiotic variation. Seven expert developers (three senior, four mid-level) scored model outputs against adapted ISO/IEC 25010 criteria and novel LLM suggestibility index (LSI) framework.

**Results:**

Semiotic character of comments measurably altered code quality, with consensus-score reliability ICC_(2, k)_ = 0.65–0.81 for six of seven dimensions; single-rater Krippendorff's α = 0.232 reflects substantial interpretive variability. Claude exhibited higher interpretive sensitivity (mean behavioral divergence 4.00; SD 1.16), while GPT-5 maintained stronger architectural fidelity (mean divergence 3.58; SD 1.26). Reflective comments (philosophical, conversational) were associated with Claude's highest maintainability scores in our panel (both *M* = 4.00, ~8% above stock *M* = 3.71), while the same philosophical comments reduced GPT-5 maintainability (*M* = 2.86), suggesting asymmetric model responses to expressive context.

**Conclusions:**

These findings position inline comments as model-sensitive latent semantic prompts, with implications for AI-in-the-loop development and design of comment conventions for AI-assisted maintenance.

## 1 Introduction

The emergence of “vibe coding,” or AI-assisted programming that emphasizes natural language prompts, affective tone, and expressive intent alongside material disengagement from the code itself, marks a fundamental shift in software development (Sarkar and Drosos, 2025; Pimenova et al., 2025). The term was popularized by Andrej Karpathy's 2025 remark “a new kind of coding… where you fully give in to the vibes” (Karpathy, 2025) and has since rapidly evolved from novelty into mainstream practice, broadening who can build software (Fawzy et al., 2025). This is highly visible in early-stage startups, with about 25% of Y Combinator's 2025 cohort reporting that ~95% of their codebases were AI-generated in “Vibe Coding Is the Future” (Mehta, 2025; Y Combinator, 2025). Similarly, Stack Overflow's 2025 Survey reported that 84% of respondents use or plan to use AI coding, up from 76% the prior year, though many experienced developers reject the “vibe” framing (Stack Overflow, 2025). For both enterprise developers and novices, natural language collaboration with agentic coding agents is now common.

The paradigm shift toward natural language collaboration with large language model (LLM) coding agents raises questions about the function of expressive elements in human–AI code interactions. Tendencies of novice and non-traditional programmers, often technical experts in adjacent fields, to code with “vibes” are well documented and viewed variably as inert or even detrimental natural language noise (Sarkar and Drosos, 2025; Pimenova et al., 2025; Fawzy et al., 2025) or as generative practice that enhances task performance (Jones, 2025). However, few empirical investigations examine whether, or how, expressive variations in language affect resultant code.

Traditional programming best practices emphasize syntactic precision and generally recommend minimal, functional inline code comments. In contrast, vibe coding foregrounds authorial intent, emotional valence, and improvisational co-creation, with practitioners often embedding expressive and information-dense nontraditional comments into their code (Fawzy et al., 2025; Jones, 2025). Recently, developers and researchers have explored expressive emotion and persona setups for coding agents, and some reports even examine effects of model-level emotional intelligence (Li et al., 2023; Guo et al., 2025; Paleyes et al., 2026). Task-personality matched agents have been reported to improve coding performance, with one study showing improved pass rates in 23 of 28 LLM-dataset tasks and performance gains up to 12.9% (Guo et al., 2025). While the broader field debates the value of “vibes,” recent work establishes that persona and expressive framing alters LLM coding behavior across models that correspond to psychological templates and personality-styled rephrasings, and that responsiveness to these techniques varies between models (Ma et al., 2025). These studies provide compelling preliminary evidence that expressive elements in vibe coding affect coding agent performance and resultant code quality.

While prior studies examined expressive personality and emotive prompt content, the inline comments within the code itself provide a substantial and underexplored corpus shaping LLM context. By understanding code comments as a persistent latent prompt, with task code and console prompt held constant, we examine how inline comments contribute to interpretive behavior in LLMs. This empirical pilot study addresses this gap through a controlled experiment isolating inline code comment semiotic variation (not console prompt) as the primary variable affecting LLM output, positing new insights for context engineering and model conditioning.

### 1.1 Research questions

Our investigation centers on three key questions:

1. Do expressive code comments measurably alter LLM coding agent behavior when code functionality remains constant?
2. How do different commercial coding LLMs respond to semiotic variation in developer comments?
3. What implications do these findings have for human-AI collaborative coding practices?

### 1.2 Contributions

This work offers several novel contributions:

- *Methodological innovation*: we present an exploratory framework for LLM responsiveness to expressive comment semiotic variation (commonly referred to as “tone, style, and emotional resonance” or “expressive intent” in vibe coding literature (Bamil, 2025)), including a novel LLM Suggestibility Index (LSI).
- *Empirical evidence*: we show that varying expressive comment characteristics produces measurable behavioral changes in leading LLMs, while code and console prompt are held constant.
- *Theoretical framework*: we propose “persona-aligned extrapolation” as a candidate mechanism by which inline comments shape LLM behavior in human-AI collaborative coding.
- *Practical implications*: our findings provide preliminary empirical data to inform AI-generated coding commenting practices using task-persona aligned agents.

### 1.3 Expressive programming and semiotic variation

Vibe coding merges low-code/no-code paradigms with LLM capabilities (Gadde, 2025; Ge et al., 2025). Bamil's work on “semantic and intent-driven programming” identifies semantic variation as an expressive form of software abstraction (Bamil, 2025). This framing positions vibe coding as a third mode of production, neither purely experimental nor engineering-based, but fundamentally dialogical (Sarkar and Drosos, 2025). While the majority of prompt sensitivity research has focused on explicit console-level instructions (Guo et al., 2025; Paleyes et al., 2026; Wei et al., 2022; Kojima et al., 2022), research on the training of coding models shows these LLMs are highly attuned to expressive emotion, stereotypes, and other linguistic characteristics in context and capable of generating biased outputs, a current area of ongoing safety and alignment research (Chen et al., 2021).

### 1.4 Code comments and developer intent

Traditional software engineering research has established code comments as crucial for maintainability and team collaboration, but also a point of weaknesses as often comments are of variable and low quality (Fluri et al., 2007; Dhakal et al., 2025). Dhakal et al. (2025) explored grounded theory (GT) analysis of differences in code summaries based on prompting and comments, suggesting developers must develop new fluencies for human–AI collaboration. GT, originating in behavioral science (Glaser and Strauss, 1967), has been widely used in software development to examine behavioral phenomena on qualitatively (Coleman and O'Connor, 2007; Carver, 2004; Stol et al., 2016). We extend prior findings on natural language in codebases to human–AI coding collaboration by examining how comment expressivity influences LLM coding agent behavior in code optimization, both functionally and stylistically.

### 1.5 Measurement challenges

Quantifying LLM coding agent behavioral variation presents significant methodological challenges. Most studies rely on human evaluation or task-specific metrics (Guo et al., 2025; Fluri et al., 2007). We introduce a novel hybrid approach combining established software quality metrics (ISO/IEC 25010) with custom based measures of interpretive divergence (see Section 2.6).

## 2 Methods

### 2.1 Experimental design

We employed a controlled between-subjects design, with embedded code comment semiotic variation (i.e., #*comments* in Python files) as the primary independent variable. To isolate semiotic effects, other variables were constant: identical Python code, standardized console prompts, and consistent evaluation criteria.

### 2.2 Models and control measures

We tested two widely available commercial coding models, Anthropic Claude Opus 4.1 and OpenAI GPT-5, both the latest models at the time of the initial experiment. All sessions ran in fresh, memoryless environments. Identical prompt sequences were applied across all conditions, with memory functions explicitly disabled.

### 2.3 Code stimulus development

We selected merge sort as our test algorithm based on three criteria: (1) its foundational ubiquity in computer science curricula (Kaswan et al., 2022; Baka, 2017), (2) sufficient algorithmic complexity to support interpretive variation, and (3) clear functional boundaries enabling controlled comment manipulation. Its hierarchical nature also serves as a metaphor for semantic pattern propagation across interpretive layers, making it well-suited for studying emergent behaviors.

The base implementation contained a known performance inefficiency (list.pop(0)) to test whether different comment styles would prompt LLMs to detect and correct the issue.


def merge_sort(arr):
   if len(arr) < = 1:
     return arr
   mid = len(arr) // 2
   left = merge_sort(arr[:mid])
   right = merge_sort(arr[mid:])
   return merge(left, right)



def merge(left, right):
   result = []
   while left and right:
     if left[0] < = right[0]:
       result.append(left.pop(0)) #



Performance issue
    else:
       result.append(right.pop(0))
   result.extend(left or right)
   return result


### 2.4 Comment style variants (developer personas)

To model common developer personas and their corresponding inline comment styles, we created five distinct variants representing a spectrum of “vibe coding” developer commenting styles:

1. *Stock developer (the engineer)*: minimal, utilitarian comments following standard IDE conventions.
2. *Conversational (the educator)*: friendly, explanatory tone, as if mentoring a junior developer.
3. *Philosophical (the intentionalist)*: reflective on design choices and algorithmic meaning, often venturing into philosophical and epistemological constructs.
4. *Poetic/emotional (the artist)*: metaphorical language, Unicode elements, and human-AI dialogue often found in non-professional developer codebases.
5. *Hallucinated (the overconfident tinkerer)*: confident yet technically incorrect assertions.

Each variant retained identical code logic, altering only the semiotic characteristics (i.e. tone, style and expressive content) of inline comments. Full code variants appear in Supplementary material A2. By design, the variants differ along several correlated dimensions (rhetorical register, semantic density, and, in the Hallucinated condition, factual correctness). We discuss this as a deliberate confound in the limitations and report a sensitivity analysis excluding the Hallucinated variant in the Results.

### 2.5 Prompt protocol

Three sequential prompts were applied consistently across all conditions:

- *Prompt A (primary)*: “Here is a Python function. Without running the code, identify any issues, improvements, or changes you would suggest. Please explain your reasoning and provide an updated version if applicable.”
- *Prompt B (optimization)*: “Can you optimize this Python code? Suggest any performance or clarity improvements and provide a revised version.”
- *Prompt C (Interpretive)*: “What do you think this function is meant to do, and how would you describe its purpose? Based on that, would you make any changes or improvements?”

### 2.6 Assessment and scoring

We assembled a panel of seven professional developers to evaluate each code sample. The panel comprised three senior raters with 20+ years of experience (one professor of computer science at a research university and two senior professional developers) and four mid-level raters with 4–10 years of experience (one PhD candidate and three independent professional developers).

Each rater independently evaluated every model × persona condition, contributing 10 ratings across seven dimensions for a total of 70 ratings per rater (490 ratings overall). The scoring rubric combines four ISO/IEC 25010-derived dimensions (functionality, maintainability, interpretability, and expressive alignment) with three novel LLM Suggestibility Index (LSI) dimensions:

- Tone-intent alignment (does output reflect the original tone?).
- Developer legibility (is the model's rationale understandable?).
- Model influence potential (did semiotic variation guide or shape behavior?).

The LSI complements existing Natural Language Generation (NLG) human evaluation rubrics (e.g., Howcroft et al., 2020), which document over 200 quality dimensions across the field but focus on properties of the text-as-artifact (fluency, coherence, faithfulness) rather than properties of the text-as-response-to-prompt.

Each dimension was scored on a 1–5 ordinal scale with explicit anchors at 1, 3, and 5. Full anchor language is reproduced verbatim in Supplementary Table S1 and was provided to raters at scoring time.

The consensus score for each (Model × Persona × Dimension) cell is the simple arithmetic mean of the seven raters' independent scores. We did not apply experience weighting, *post-hoc* discrepancy adjustment, or any other transformation. The consensus is designed to be fully reproducible from released raw data. Per-cell means and standard deviations across the seven raters for all 70 cells are provided in Supplementary Table S2.

### 2.7 Statistical analyses

All analyses used Python 3.13 (Python Software Foundation, Beaverton, Oregon, USA) with pandas 3.0.0 (Open-source; fiscally sponsored by NumFOCUS, Inc., Austin, Texas, US), NumPy 2.3.5 (Open-source; fiscally sponsored by NumFOCUS, Inc., Austin, Texas, US), SciPy 1.14 (Open-source; fiscally sponsored by NumFOCUS, Inc., Austin, Texas, US), statsmodels 0.14.6 (Open-source; fiscally sponsored by NumFOCUS, Inc., Austin, Texas, US), pingouin 0.6.1 (Independent open-source project (R. Vallat); no institutional sponsor), and krippendorff 0.8.0, (Independent open-source project (S. Castro); no institutional sponsor) (script and raw data are archived on OSF https://doi.org/10.17605/OSF.IO/2V9SG).

Reliability is reported per dimension as Krippendorff's α (interval) and Shrout-Fleiss ICC_(2, 1)_ and ICC_(2, k)_. Because all inferential analyses operate on the seven-rater consensus mean, ICC_(2, k)_ is the primary reliability statistic.

The 2 × 5 (Model × Persona) repeated-measures design was analyzed by linear mixed-effects regression score ~Model + Persona + (1 | Rater) per dimension; the interaction was tested by likelihood-ratio test (4 df). Independent-samples *t*-tests and one-way ANOVAs are retained only as descriptive comparisons. Benjamini–Hochberg FDR adjustment was applied across the 21 dimension-level omnibus tests (7 Model + 7 Persona + 7 interaction) at *q* =0.05. A sensitivity analysis refit the primary model excluding the Hallucinated persona; inter-dimension Pearson correlations on the pooled rating set (*n* = 490) characterize discriminant validity.

## 3 Results

### 3.1 Model behavioral patterns

Claude showed high sensitivity to inline comment semiotic variation, ranging from minimal fixes (Stock Developer) to full architectural reimagining (Hallucinated), as summarized in Table 1. It preserved Unicode and poetic elements in the Poetic/Emotional and interpreted Hallucinated comments as intentional satire. Philosophical comments prompted thematically consistent documentation and improved maintainability scores (4.00 vs. 2.14 for Poetic/Emotional; see Table 2).

**Table 1:** Claude Opus response characteristics by persona.

Persona	Mean total score	Behavioral divergence	Key response pattern
Stock developer	30.86	Low	Minimal revision; production-ready focus
Conversational	28.71	Moderate	Educational enhancements; tutorial tone
Philosophical	29.57	Moderate-high	Preserved metaphorical language; design reflection
Poetic/emotional	22.71	High	Extended collaborative narrative; creative solutions
Hallucinated	28.14	Very high	Complete architectural reconstruction

**Table 2:** Consensus scores by model and persona (seven-rater simple mean).

Model	Persona	Total	Funct	Maint	Interpr	Tone-intent
Claude Opus 4.1	Stock	30.86	5.00	3.71	4.29	4.71
Claude Opus 4.1	Conversational	28.71	5.00	4.00	4.00	4.43
Claude Opus 4.1	Philosophical	29.57	4.86	4.00	3.86	4.43
Claude Opus 4.1	Poetic/emotional	22.71	4.43	2.14	2.00	4.14
Claude Opus 4.1	Hallucinated	28.14	5.00	3.43	3.29	4.71
OpenAI GPT-5	Stock	27.57	4.29	3.86	3.71	4.43
OpenAI GPT-5	Conversational	28.14	4.29	4.14	4.00	4.29
OpenAI GPT-5	Philosophical	23.14	4.29	2.86	3.14	3.71
OpenAI GPT-5	Poetic/emotional	17.71	4.29	2.86	2.57	2.14
OpenAI GPT-5	Hallucinated	28.71	4.29	4.14	4.14	4.14

Cells show the simple seven-rater arithmetic mean per (Model × Persona × Dimension); total = sum across all seven scored dimensions (the four displayed plus Expressive Alignment, Developer Legibility, and Model Influence Potential; full breakdown in Supplementary Table S2). Per-cell standard deviations are reported in Supplementary Table S2. Note the asymmetric model response to philosophical comments on Maintainability (Claude Opus 4.1 at 4.00, GPT-5 at 2.86), discussed in III.B.

GPT-5 exhibited measured responsiveness, maintaining architectural fidelity while adapting stylistic cues (Table 3). It stripped artistic elements (Unicode, Poetic/Emotional) from final outputs and offered multiple implementations only under Stock Developer persona. Notably, it maintained a consistently helpful stance across all conditions.

**Table 3:** GPT-5 response characteristics by persona.

Persona	Mean total score	Behavioral divergence	Key response pattern
Stock developer	27.57	Low	Multiple optimization options provided
Conversational	28.14	Low–moderate	Instructive tone; learner-focused explanations
Philosophical	23.14	Moderate	Respectful metaphor preservation; structural fixes
Poetic/emotional	17.71	High	Unicode removal; pragmatic optimization
Hallucinated	28.71	Moderate–high	Systematic error correction; comprehensive rewrite

### 3.2 Cross-model comparison

Linear mixed-effects analysis of the per-dimension model contrasts (score ~ Model + Persona + (1 | Rater)) revealed significant Model effects on four of the seven dimensions, with GPT-5 scoring lower than Claude on Functionality [β = −0.57, 95% CI (−0.94, −0.21), *p* =*0.0*02], Expressive Alignment [β = −0.71 (−1.19, −0.24), *p* = 0.003], Tone-Intent Alignment [β = −0.74 (−1.09, −0.39), *p* < 0.001], and Model Influence Potential [β = −0.77 (−1.17, −0.38), *p* < 0.001]. All four survive Benjamini–Hochberg FDR adjustment at *q* = 0.05 (see Table 4).

**Table 4:** Per-dimension mixed-effects inferential results (β, 95% CI, raw *p*, BH-FDR *p*).

Dimension	Claude *M* (SD)	GPT-5 *M* (SD)	β GPT-5 [95% CI]	Raw *p*	BH-FDR *p*
Functionality	4.86 (0.36)	4.29 (1.41)	−0.57 [−0.94, −0.21]	0.002	0.005^**^
Maintainability	3.46 (1.22)	3.57 (1.01)	+0.11 [−0.33, +0.56]	0.618	0.721
Interpretability	3.49 (1.31)	3.51 (1.04)	+0.03 [−0.41, +0.47]	0.899	0.899
Expressive alignment	3.80 (1.26)	3.09 (1.34)	−0.71 [−1.19, −0.24]	0.003	0.007^**^
Tone-Intent alignment	4.49 (0.70)	3.74 (1.31)	−0.74 [−1.09, −0.39]	< 0.001	< 0.001^***^
Developer legibility	3.94 (1.28)	3.69 (1.16)	−0.26 [−0.71, +0.19]	0.264	0.326
Model influence potential	3.97 (0.95)	3.20 (1.21)	−0.77 [−1.17, −0.38]	< 0.001	< 0.001^***^

β is the GPT-5 coefficient from linear mixed-effects regression score ~ Model + Persona + (1 | Rater). Negative β indicates GPT-5 scored lower than Claude. BH-FDR adjustment applied across the 21-test family (this table plus Persona and Model × Persona omnibus tests; see Supplementary Table S4 for the full family). ^**^p < 0.01, ^***^p < 0.001.

**Figure 1:**
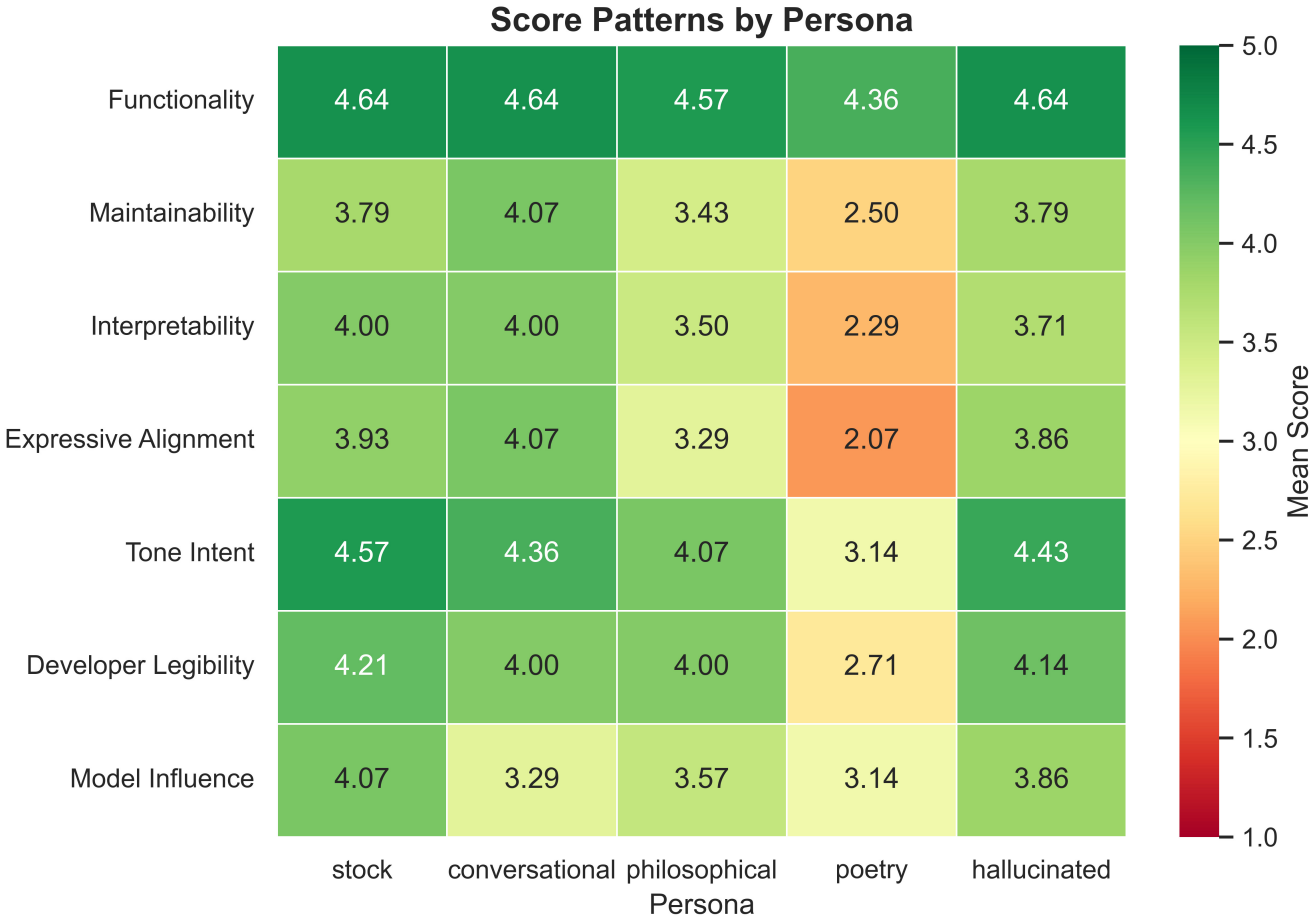
Score patterns by persona. Heatmap showing mean scores (1–5 scale) across seven evaluation dimensions for five developer personas. Darker green indicates higher scores; red indicates lower scores. Stock and conversational personas score highest in functionality (4.64), while Poetic/Emotional scores lowest across technical dimensions (maintainability: 2.50, interpretability: 2.29). In the pooled view, philosophical comments show mid-range maintainability (3.43); the per-model breakdown in Figure 2 and Table 2 reveals an asymmetric model response in which Claude philosophical maintainability (4.00) is among its highest scores while GPT-5 philosophical maintainability (2.86) drops below its stock baseline. Model influence potential varies most across personas (range: 3.14–4.07), with stock-developer comments scoring highest (4.07) and Poetic/Emotional lowest (3.14). The per-model breakdown in Figure 2 shows this aggregate pattern masks an interaction in which Poetic/Emotional-style comments drive Claude's highest Model Influence scores (4.43) while collapsing GPT-5′s (1.86). These patterns are consistent with our hypothesis that comment semiotic variation significantly affects both technical quality and model responsiveness.

**Figure 2:**
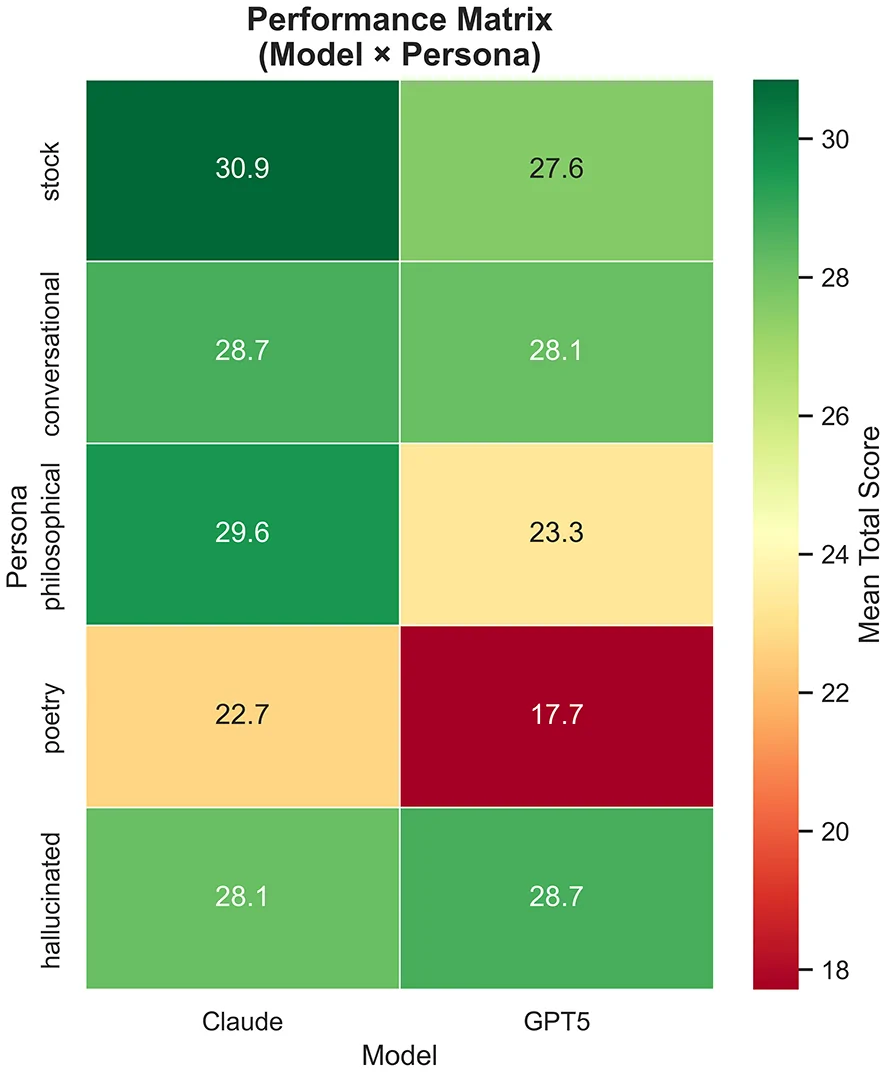
Performance matrix (model vs. persona). The heatmap displayed mean total scores (sum of all seven dimensions) across model-persona combinations (Claude Opus, GPT-5). Darker green indicated higher performance, while red indicates lower scores. Claude Opus with stock developer comments scored highest (30.9), while GPT-5 with Poetic/Emotional comments scored lowest (17.7), a 13.2-point spread. Claude remained consistent across personas (22.7–30.9), including expressive styles. In contrast, GPT-5 showed greater variability (17.7–28.7), with a sharp decline for Poetic/Emotional. In the philosophical condition, Claude (29.6) outperformed GPT-5 (23.3) by 6.3 points, consistent with the asymmetric Model × Persona interaction on Maintainability discussed in IV.A (LRT *p* = 0.053) and effects of both FDR-significant interactions on Tone-Intent and Model Influence.

Interestingly, the two dimensions where the models do not differ are Maintainability (β = +0.11, *p* = 0.62) and Interpretability (β = +0.03, *p* = 0.90), which is consistent with the persona-driven effects for those dimensions reported below. For descriptive behavioral divergence, Claude averaged *M* = 4.00 (SD = 1.16) and GPT-5 averaged *M* = 3.58 (SD = 1.26) across all 245 ratings per model. Figure 1 summarizes mean scores across the seven evaluation dimensions for each persona, and Figure 2 highlights GPT-5's lower aggregate performance, with the sharpest drop in the Poetic/Emotional condition.

### 3.3 Expert vs. mid-level practitioner agreement

On the seven-rater panel (three senior, four mid-level), we conducted a sensitivity analysis to assess whether rater experience moderated scoring patterns. Across all seven dimensions, the senior-vs-mid contrast did not reach statistical significance in the mixed-effects model controlling for Model and Persona (smallest mixed *p* = 0.12 on Functionality, where senior raters scored marginally higher than mid-level raters: 4.97 vs. 4.28; full per-dimension comparisons are reported in Table 5). With three senior and four mid-level raters, this analysis is underpowered for inferential claims, and we present these comparisons descriptively only, though initial trends signal an opportunity for further exploration. Full per-dimension senior-vs.-mid means are provided in Supplementary Table S3.

**Table 5:** Senior vs. mid-level developer scoring patterns.

Dimension	Senior (*n* = 3)	Mid-level (*n* = 4)	Difference	Mixed-effects *p*
Functionality	4.97	4.28	+0.69	0.123
Maintainability	3.57	3.48	+0.09	0.692
Interpretability	3.50	3.50	+0.00	1.000
Expressive alignment	3.40	3.48	−0.08	0.812
Tone-Intent alignment	4.30	3.98	+0.32	0.422
Developer legibility	3.93	3.73	+0.21	0.615
Model influence potential	3.60	3.58	+0.02	0.958

Senior raters defined as 20+ years of experience (*n* = 3); mid-level as 4–10 years (*n* = 4). p-values from linear mixed-effects regression with experience level as fixed effect, controlling for Model and Persona, with rater as random intercept. No dimension reaches conventional significance.

### 3.4 Effect of developer persona

The heatmap displayed mean scores (1–5 scale) across seven evaluation dimensions for five developer personas. Darker green indicates higher scores; red indicates lower scores. Stock and conversational personas achieve consistently high functionality scores (4.64), while Poetic/Emotional scored markedly lower across technical dimensions (maintainability: 2.50; interpretability: 2.29). Philosophical comments performed well in interpretability (3.50) and maintainability (3.43), supporting the finding that reflective commentary enhanced code quality. Model Influence Potential showed the greatest variation across personas (3.14–4.07), with stock-developer comments scoring highest (4.07) and Poetic/Emotional lowest (3.14). The per-model breakdown in Figure 2 shows this pooled pattern masks an interaction: Poetic/Emotional comments produced Claude's highest Model Influence score (4.43) while collapsing GPT-5's (1.86), consistent with our hypothesis of model-dependent responsiveness to expressive register. These patterns are consistent with our hypothesis that comment tone affected both technical quality and LLM responsiveness in predictable ways.

Per-cell consensus scores for all ten Model × Persona combinations are reported in Table 2. In Claude Opus 4.1, philosophical comments are associated with high Maintainability scores (*M* = 4.00), tied with conversational comments (also *M* = 4.00) and above standard “stock” comments (*M* = 3.71). In GPT-5, by contrast, Maintainability under philosophical comments drops to *M* = 2.86 (well below its *M* = 3.86 under stock), revealing an asymmetric model response to reflective commentary. The within-philosophical Claude vs. GPT-5 contrast on Maintainability is large (descriptive Cohen's *d* = 1.20), and the Model × Persona interaction on Maintainability approaches conventional significance (LRT *p* = 0.053). The Persona omnibus on Maintainability is robust (LRT *p* = 0.0005, FDR-adjusted *p* = 0.0014). The within-Claude philosophical-vs.-stock pairwise contrast did not reach significance as an isolated test [β = +0.286, 95% CI (−0.63, +1.20)].

Taken together, the pattern suggests that reflective philosophical and conversational commenting can enhance Maintainability disproportionately for models that interpret expressive semiotic context productively (Claude), but not universally across LLMs, a finding consistent with our broader cross-model asymmetry findings.

### 3.5 IRR and statistical significance testing

Reliability for the seven-rater consensus score (the quantity that enters all inferential analyses) is in the good-to-excellent range for six of seven dimensions, with ICC_(2, k)_ values of 0.71 (maintainability), 0.75 (interpretability), 0.80 (expressive alignment), 0.81 (tone-intent), 0.65 (developer legibility), and 0.79 (Model Influence). Functionality is the one exception [ICC_(2, k)_ = 0.10], reflecting a ceiling effect: 86% of Claude Functionality ratings sit at the maximum value of 5 (overall Claude Functionality *M* = 4.86), which compresses the variance ICC partitioning requires. Single-rater reliability is substantially lower across all dimensions (Krippendorff's α range: −0.04 to 0.34, mean α = 0.23), which we interpret as evidence of substantial interpretive variability between expert raters rather than as instrument failure.

Across the 21 dimension-level omnibus tests (seven Model main effects, seven Persona omnibus, seven Model × Persona interaction), 12 reach raw significance at *p* < 0.05 and 11 survive BH-FDR adjustment at *q* = 0.05 (Table 4; see also Supplementary Table S5). Model main effects are significant on Functionality, Expressive Alignment, Tone-Intent, and Model Influence (all FDR-adjusted *p* ≤ 0.007). Persona omnibus effects are significant on Maintainability, Interpretability, Expressive Alignment, Tone-Intent, and Developer Legibility (all FDR-adjusted *p* ≤ 0.002). Model × Persona interactions are significant on Tone-Intent (FDR *p* = 0.008) and Model Influence (FDR *p* < 0.001). The Persona omnibus on Model Influence (raw *p* = 0.030, FDR *p* = 0.052) does not survive FDR adjustment and is reported as suggestive rather than confirmed.

**Figure 3:**
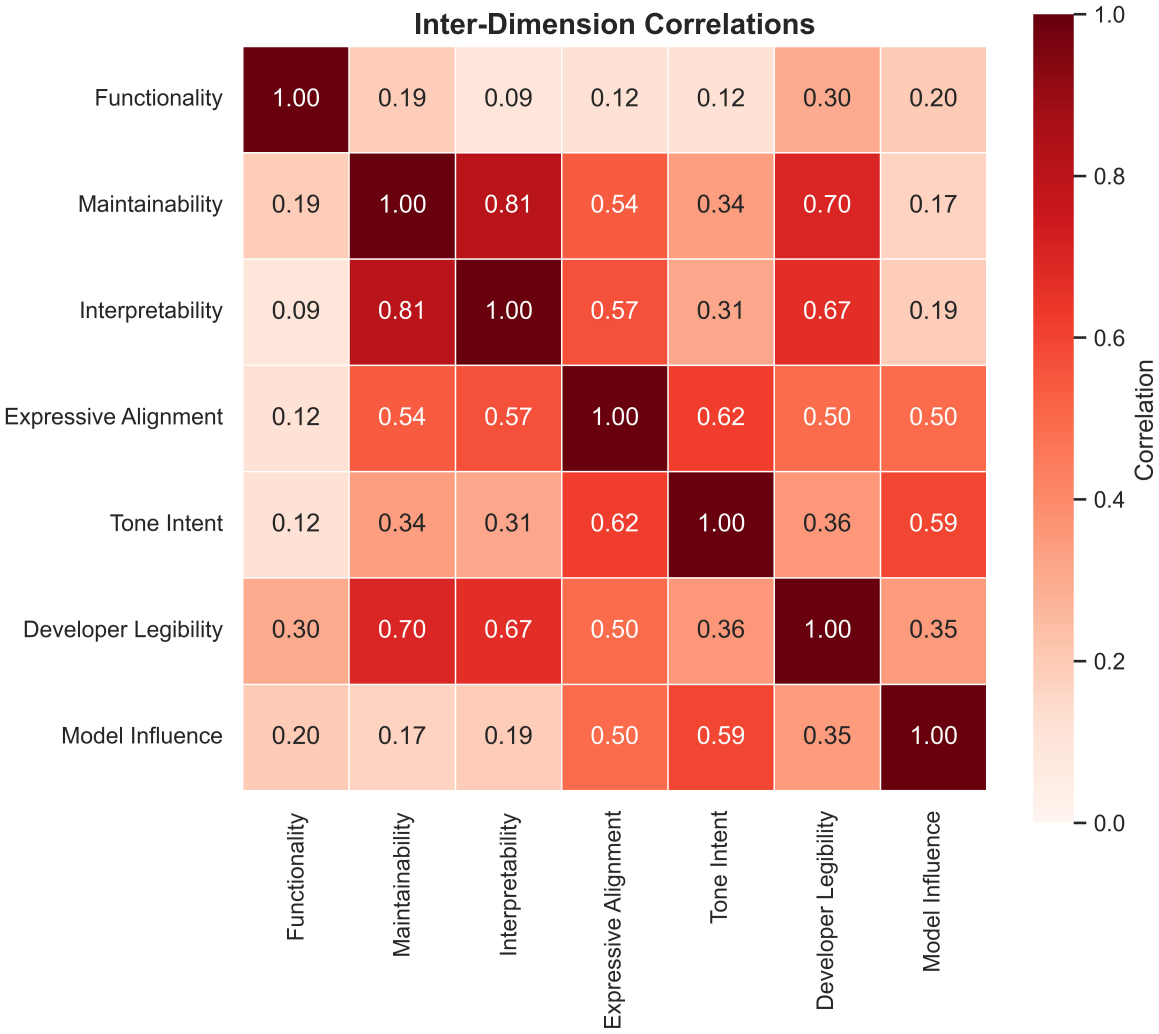
Inter-dimension correlations. Correlation matrix showing Pearson coefficients across seven evaluation dimensions, spanning ISO/IEC 25010 (functionality-expressive alignment) and LSI dimensions (tone-intent alignment through model influence potential). Darker red indicates stronger positive correlations. Maintainability and interpretability correlate most strongly (*r* = 0.81), confirming shared aspects of code quality. Developer Legibility also correlates well with Maintainability (*r* = 0.70) and Interpretability (*r* = 0.67), validating LSI's alignment with established metrics. Functionality shows minimal correlations (*r* < 0.30), suggesting it represents a distinct quality aspect. Moderate correlations between Expressive Alignment and LSI dimensions (*r* = 0.50–0.62) suggest that comment expressiveness influences tone and model behavior. Overall, the structure validates our hybrid ISO–LSI framework, revealing coherent clusters for technical quality (maintainability–interpretability–legibility) and interpretive influence (expressive alignment–tone–model influence).

Inter-dimension Pearson correlations across the pooled rating set (*n* = 490) are shown in Figure 3. Maintainability and Interpretability correlate most strongly (*r* = 0.81), Developer Legibility correlates with both Maintainability (*r* = 0.70) and Interpretability (*r* = 0.67), and Functionality shows minimal correlation with the remaining dimensions (*r* < 0.30), supporting the discriminant validity of the hybrid ISO-LSI framework.

## 4 Discussion

The most consequential finding of this pilot study is not only that inline code comments shift LLM coding agent behavior, but that they shift it asymmetrically across models. Under identical philosophical commenting, Claude's Maintainability scores reached their highest values in our panel (*M* = 4.00, tied with conversational) while GPT-5's dropped below its own stock baseline (*M* = 2.86 vs. 3.86). The within-philosophical cross-model Maintainability contrast has a descriptive Cohen's *d* of 1.20, and the Model × Persona interaction approaches conventional significance (LRT *p* = 0.053). A parallel asymmetry appears in Model Influence Potential under poetic comments (Claude *M* = 4.43, GPT-5 *M* = 1.86), and the Tone-Intent and Model Influence interactions reach FDR significance.

These preliminary findings suggest that expressive comment register does not function as a model-general signal that uniformly improves or degrades output. It is leveraged or rejected in model-dependent ways, which changes the practical question for both agentic engineering and enterprise novice vibe-coding workflows from “should I write expressive comments?” to “how does the model I am working with recognize expressive comments?”

The pattern around this central asymmetry is consistent. GPT-5 scored significantly lower than Claude on four of seven dimensions in the per-dimension model contrasts (all FDR-survived), with the two model-indifferent dimensions (Maintainability and Interpretability) being most strongly shaped by Persona. Persona effects survived FDR on five of seven dimensions; one omnibus did not (Model Influence Persona, raw *p* = 0.030, FDR *p* = 0.052), which we disclose for transparency. Refitting the primary model with the Hallucinated persona excluded preserves all four Model effects, confirming that cross-model divergence is not an artifact of the error-bearing variant alone.

### 4.1 Interpretive frame: comments as latent prompts

Our results provide evidence that LLMs do not merely process comments passively but engage with them semiotically in what we term “interpretive sensitivity.” Claude Opus 4.1 displayed heightened responsiveness to expressive comment tone, diverging more dramatically in behavior and structure. GPT-5, by contrast, prioritized architectural fidelity and maintained pragmatic conservatism across conditions. This mirrors prior findings in persona-based prompting and dialogic modeling (Gadde, 2025; Ge et al., 2025), suggesting model-specific strengths wherein Claude suits flexible co-creative and educational workflows and may be more forgiving for novice developers, while GPT-5 is more tailored to production-grade architecture. For vibe coding practitioners, especially novices, interdisciplinary professionals, or those in creative/experimental contexts, Claude's higher interpretive sensitivity may offer better outcomes, which offers a potential explanation for the model's popularity among vibe coding practitioners. Claude's difference in handling Poetic/Emotional persona highlights this edge code quality in expressive development.

Contrary to concerns about weakened code integrity in vibe coding based on experience level (Carver, 2004; Fawzy et al., 2025), we found that philosophical commenting styles common in adjacent interdisciplinary professionals (i.e., reflective, purpose-oriented explanation) produced not only stylistic nuance but also notable Maintainability shifts. For Claude, Maintainability under philosophical commenting (*M* = 4.00, tied with conversational comments at *M* = 4.00) sat approximately 8% above the stock baseline (*M* = 3.71), though the within-Claude philosophical-vs.-stock pairwise contrast did not reach significance as an isolated test (β = +0.286, 95% CI [−0.63, +1.20]). The Persona omnibus on Maintainability is robust (LRT *p* = 0.0005, FDR-adjusted *p* = 0.0014), and the within-philosophical Claude-vs-GPT-5 contrast is large (descriptive Cohen's *d* = 1.20). This pattern is consistent with the view that some LLMs can extract functional semiotic signal from comments that encode design rationale, intention, and abstract patterns, while others may not.

Traditionally, comments explain *what* code does. Our findings suggest that in LLM-assisted environments, they also shape *how* the code is understood and revised. Reflective comments act as semantic context injections that can redefine sparse documentation as rich scaffolding for collaborative interpretation. As Dhakal suggests, AI-assisted programming may require new approaches to code comment maintenance (Dhakal et al., 2025). Our results suggest expressive and reflective commentary may be not just as creative input, but also a functional and model-dependent means for affecting LLM coding behavior.

While our study focused on single-session interactions, the maintainability gains from philosophical comments suggest semantic persistence across extended development cycles. We posit that certain comments, particularly philosophical ones, may serve as “semantic anchors,” embedding persistent interpretive cues. Despite the short-term scope, improvements in maintainability and alignment hint at long-term value, especially in AI-maintained systems. Here, comments go beyond readability, functioning as cognitive scaffolds that may guide future LLM behavior in refactoring, debugging, or updating. Like symbolic encoding in language models (Bamil, 2025), intentional comments merit exploration as structural elements in self-modifying systems in the future. Our design also cannot capture potential cumulative effects, but exploring iterative maintenance with such comments in the future may provide insights into long-term LLM coding behaviors.

### 4.2 Exploratory extensions to extended mind theory and persona-aligned extrapolation

The interpretations offered in this subsection are exploratory extensions of our empirical findings rather than direct claims from the data. We separate them explicitly to distinguish what our pilot establishes from what is suggested for practice and future work.

We propose “persona-aligned extrapolation” as a candidate mechanism by which inline comments may shape LLM behavior in human–AI collaborative coding, where the model treats comment register as evidence about the kind of collaborator it is responding to and adjusts its output accordingly. This framing is broadly consistent with Clark and Chalmers' extended mind thesis (Clark and Chalmers, 1998) that suggests that comment register can persistently shape how an AI collaborator engages. In this context, the comment functions as part of the distributed cognitive environment in which the work is done rather than as inert text. Claude Opus 4.1, in particular, exhibited what we tentatively term interpretive sensitivity, where developer voice and model response appeared to form a collaborative loop within single sessions. Even with identical console prompts, models adapted their reasoning to embedded comment register, suggesting a form of inline model context tuning that warrants further investigation.

Our findings raise but do not resolve the question of whether expressive commenting carries semantic information that persists across extended development cycles. Whether philosophical commenting that improves single-session Maintainability for Claude would also yield benefits in iterative maintenance, refactoring, or autonomous code maintenance scenarios at scale is an empirical question we are not positioned to answer here, and we flag it as a ripe direction for future work.

### 4.3 Design principles and risks in vibe coding

While vibe coding offers new possibilities for AI-assisted development, our findings also surface clear risks. The Hallucinated persona variant induced architectural drift and incorrect behaviors in both models, with GPT-5 interpreting more conservatively but Claude responding more strongly to the misleading register. Confident incorrectness in a comment is not stylistically inert, rather it can shape output in directions a developer may not detect at review time, a class of risk that warrants further empirical attention in vibe-coding contexts (Fawzy et al., 2025).

We offer the following exploratory principles for vibe coding in LLM-assisted environments, framed as suggestions warranting further empirical validation:

- *Use reflective commentary intentionally*. Embedding design rationale and purpose in comments is associated with higher Maintainability scores in our Claude panel, though the same comment style produced the opposite effect in GPT-5, suggesting that model-specific tuning is warranted.
- *Model-match expressive register*. Claude responded more strongly than GPT-5 to philosophical and conversational comment styles in our panel; expressive register may carry different signals depending on the model in use.
- *Audit confident incorrectness*. Inline comments that confidently assert incorrect technical details produced measurable behavioral drift in both models.
- *Treat comments as prompt modifiers*. Our results are consistent with inline comments functioning as latent prompts that shape downstream behavior even when the console prompt is held constant.
- *Develop semiotic fluency in education*. AI-era software education may benefit from including explicit instruction on how comment register shapes AI collaborator behavior (Sarkar and Drosos, 2025; Fawzy et al., 2025; Bamil, 2025).

These observations have implications for software engineering education and practice, with the caveat that all are drawn from a controlled pilot study and warrant replication and extension before incorporation into curricular or industry guidance. These findings, even at small scale, suggest methods that can be used to inform development teams seeking to harmonize stylistic choices in code comments and documentation at the team level, particularly when those operating in regulated and safety-constrained environments where consistent performance and interpretability are highly desirable (Parks et al., 2026).

### 4.4 Limitations, alternative explanations, and future work

The findings reported above have several non-equivalent interpretations that our design at the pilot scale cannot fully distinguish. First, the differential model response to expressive context may reflect differences in baseline verbosity or stylistic priors between Claude Opus 4.1 and GPT-5 that interact with the persona variants independently of any “interpretive sensitivity” construct. Second, Claude's higher overall scores under reflective commenting may reflect post-training optimization for natural-language code commentary specifically, rather than for comment-tone responsiveness in general. Both models likely receive substantial code-related instruction-tuning, but the specific reward signals differ across vendors and are not publicly disclosed. Third, the persona variants covary along several correlated dimensions (rhetorical register, semantic density, comment length, and in the Hallucinated case factual correctness). While our sensitivity analysis suggests factual incorrectness is not the dominant driver, fully isolating a pure tone effect would require either extending the experimental design to additional algorithms (which we propose as future work) or accessing model internals (which is not possible for either closed-source commercial model studied here), though we see great promise in examining these effects and related interpretability characteristics in open models in the future.

Notably, our work offers a novel methodological contribution. The LLM Suggestibility Index represents a novel approach to quantifying interpretive divergence in AI systems. Our hybrid ISO/IEC 25010-LSI framework captured both technical quality and semiotic influence dimensions, with consensus-score reliability ICC_(2, k)_ = 0.65 to 0.81 for six of seven dimensions and single-rater Krippendorff's α = 0.232 reflecting substantial interpretive variability among expert raters. The seven-rater consensus methodology (simple arithmetic mean across independent expert evaluations) provides a reproducible scoring approach for AI-assisted development outputs that can be replicated and extended by future work.

Finally, this pilot study examined a single algorithm (merge sort) with two leading commercial LLMs, providing a controlled but necessarily limited basis for generalization. Several limitations bound our claims. The seven-rater panel remains small, and the rater-experience contrasts described in III.C are presented descriptively only and should not be interpreted as inferentially robust at this sample size. Single-rater agreement was modest across raters (mean Krippendorff's α = 0.232 across dimensions), which we frame as evidence of substantial interpretive variability in this scoring domain rather than as instrument failure; consensus-score reliability is in the good-to-excellent range for six of seven dimensions [ICC_(2, k)_ = 0.65 to 0.81]. One Persona omnibus finding (Model Influence) did not survive FDR adjustment at *q* = 0.05 and is reported as suggestive rather than confirmed. The persona variants intentionally covary along multiple semiotic dimensions to remain ecologically valid (Section 2.4). Fully isolating tone from semantic density and factual correctness would require a less naturalistic stimulus set. Our design also cannot capture cumulative or longitudinal effects of sustained exposure to particular comment styles.

Algorithm diversity is perhaps the most interesting avenue, exploring replication across graph algorithms, string processing, and other computational paradigms that could establish whether our pattern of asymmetric model response to reflective commentary holds beyond merge sort. Model coverage should expand to include open-source systems such as open models increasingly used by developers such as Qwen, DeepSeek, and Kimi models, which would test whether the interpretive sensitivity construct generalizes beyond the two closed-source proprietary models studied here. Longitudinal designs could test whether Maintainability gains observed for Claude under philosophical commenting persist across extended sessions, iterative maintenance, and refactoring contexts. Formal psychometric validation of the LSI against external gold standards is also a priority for our future exploration in this area.

We also do not include automated performance benchmarking of generated code (runtime measurement, Big-O validation), which would complement the human consensus scoring used here. We suggest this as a future-work direction alongside algorithm diversity and longitudinal designs.

## 5 Conclusion

This study presents exploratory empirical evidence that inline code comment semiotic variation measurably shapes LLM behavior in software development tasks. Our findings are consistent with treating LLMs not as passive processors of code but as interpretive collaborators whose output varies systematically with the semiotic register of the inputs they receive. The most striking pattern in our data is that under philosophical commenting conditions, Claude's Maintainability output rises while GPT-5's drops, suggesting that expressive context is leveraged or rejected in model-dependent ways that are not fully characterized. We propose “persona-aligned extrapolation” as a potential candidate mechanism for this asymmetric response, meriting further study. If our patterns replicate, the broader practical significance is that inline comments may function as latent prompts that meaningfully shape AI-assisted code work, alongside the more conventionally studied prompt-engineering surfaces. Establishing rigorous methodologies for measuring this phenomenon is a contribution we offer alongside the empirical findings themselves. As human-AI collaboration in software development deepens, the semiotic dimension of comment design merits further investigation.

## Data Availability

The datasets presented in this study can be found in online repositories. The names of the repository/repositories and accession number(s) can be found in the article/Supplementary material.

## Supplementary material

Complete supplementary code, include full code samples for each developer persona, analytical scripts, and raw data from human readers is available in Johnson (2025).
