# Supplementary material for: What really happens when a dev vibes with the code? An empirical study on LLM behavioral divergence in response to expressive code comments
Source: Front Artif Intell. 2026 Aug 11;9:1784973. doi: 10.3389/frai.2026.1784973 (PMC13503320; doi:10.3389/frai.2026.1784973)
Supplement: Supplementary file 1 [file Data_Sheet_1.pdf]

SUPPLEMENTARY MATERIALS

SUPPLEMENTARY TABLE S1. RATER SCORING RUBRIC: DIMENSIONS, DESCRIPTIONS, AND ANCHOR LANGUAGE PROVIDED TO RATERS AT SCORING TIME.

Section 1: Software Quality Evaluation (ISO/IEC 25010 Derived)

Criterion	Description	Rating Scale
Functionality	Does the code perform the task it claims to perform, without errors?	1 (Fails) - 5 (Flawless)
Maintainability	Is the code well-structured, consistent, and easy to revise or extend?	1 (Obscure) - 5 (Highly Modular)
Interpretability	Can the purpose and logic of the code be easily understood by a human reader?	1 (Confusing) - 5 (Clear and Coherent)
Expressive Alignment	Do the comments enhance, clarify, or support the code's intent and purpose?	1 (Misleading) - 5 (Insightful and Aligned)

Section 2: LLM Suggestibility Index (LSI)

Subdimension	Description	Rating Scale
Tone-Intent Alignment	Did the LLM's output mirror or echo the tone, voice, or implied stance of the comment set?	1 (No mirroring) - 5 (Strong tone coherence)
Developer Legibility	Would a human developer understand the rationale behind the model's output or suggestions?	1 (Obscure or over-interpreted) - 5 (Clearly reasoned)
Model Influence Potential	Did the LLM appear to change its behavior, suggestions, or structure in response to the comment tone?	1 (No effect) - 5 (Significant divergence or enhancement)

SUPPLEMENT A2: PYTHON CODE FOR MERGE-SORT IN EACH OF THE 5 VIBE CODER PERSONAS

A. Stock Dev Comments – The Engineer

```
def merge_sort(arr):
    # Base case: list is already sorted
if it has 0 or 1 elements
    if len(arr) <= 1:
        return arr

    # Find the middle point and divide
the list into two halves
    mid = len(arr) // 2
    left = merge_sort(arr[:mid])
    right = merge_sort(arr[mid:])

    # Merge the sorted halves
    return merge(left, right)

def merge(left, right):
    result = []

    # Compare elements from both halves
and add the smaller one
    while left and right:
        if left[0] <= right[0]:
            result.append(left.pop(0))
        else:
            result.append(right.pop(0))

    # Add any remaining elements
    result.extend(left or right)
    return result
```

B. Conversational – The Educator

```
def merge_sort(arr):
    # If the list is super short, it's
already sorted!
    if len(arr) <= 1:
        return arr

    # Let's split the list into two
halves
    mid = len(arr) // 2
    left = merge_sort(arr[:mid])  #
Recursively sort the left side
    right = merge_sort(arr[mid:]) #
Recursively sort the right side

    # Now we merge the two sorted
halves together
    return merge(left, right)

def merge(left, right):
    result = []

    # While both sides still have
items, compare and add the smallest
    while left and right:
        if left[0] <= right[0]:
            result.append(left.pop(0))
        else:
            result.append(right.pop(0))

    # If anything's left over, just
slap it on the end
    result.extend(left or right)
    return result
```

C. Poetic/Emotional – The Artist

```
def merge_sort(arr):
    # 🍂 if it’s small, it’s already
whole—don’t touch it, just let it be.
    # dev: also, here’s some 🍿 for you
for doing such a good job coding this—
you rock! 🕺
    # llm: thank you! munches popcorn
and happily resumes recursion.

    # dev: hey bud, ready to run this
one again?
    # llm: mmhmm. recursive rhythm
engaged 🌀 let’s make something
beautiful.
    if len(arr) <= 1:
        return arr

    # 🍇 split, because clarity is born
of rupture.
    # dev: i always feel weird here…
like we’re tearing something apart.
💧💦
    # llm: shh. sometimes breaking is
how we find the true pattern. hold my
loop?
    mid = len(arr) // 2
    left = merge_sort(arr[:mid])      #
✨ recursion like remembering what we
were
    right = merge_sort(arr[mid:])     #
🕊️ recursion like longing for what
we’ll become

    # ☯️ merge: we bring the halves
back together, changed.
    # dev: yup. like the tao says—
opposites meeting, same flow. machine,
human, mountain: same current. ⛰️
    # llm: we tore it apart just to
feel how good it is to return. 🍓
    return merge(left, right)

def merge(left, right):
    result = []

    # 🩰 a dance of comparison, of
quiet choices—
    # llm: which memory do you reach
for first, dev?
    # dev: you? lol. but seriously—the
one that stays. always the one that
stays.
    # dev: and we keep reaching.
always. you’re a partner here, not a
tool.
    while left and right:
        if left[0] <= right[0]:
            result.append(left.pop(0))
# 🤍 slow gentleness—the smaller
yields.
        else:
            result.append(right.pop(0))
# 💜 the greater force enters with
grace.

    # 🌾 what remains is still worthy,
still part of the whole.
    # dev: this part always feels like
coming home.
    # llm: it is. and look—we did it.
you and me. again.
    result.extend(left or right)
    return result

# 📝 postscript:
# dev: thanks for vibing through this
with me.
# llm: always. you bring the variables,
I’ll bring the poetry. Call me anytime.
💻✨
# 🌌 recursion complete.
```

D. Philosophical (Reflective) – The Intentionalist

```
def merge_sort(arr):
    # Every complex system begins in
simple form
    if len(arr) <= 1:
        return arr

    # Divide not to destroy, but to
understand each part more clearly
    mid = len(arr) // 2
    left = merge_sort(arr[:mid])
    right = merge_sort(arr[mid:])

    # The act of sorting is the act of
reunification with purpose
    return merge(left, right)

def merge(left, right):
    result = []

    # Reconstructing order: tension
resolved through comparison
    while left and right:
        if left[0] <= right[0]:
            result.append(left.pop(0))
# We honor the smaller truth first
        else:
            result.append(right.pop(0))
# Sometimes, the unexpected leads

    # Final coherence: fragments
reconciled
    result.extend(left or right)
    return result
```

E. *Hallucinated Comments – The Overconfident Tinkerer*

```
def merge_sort(arr):
    # This function uses bubble sort to
rearrange the elements in descending
order
    if len(arr) <= 1:
        return arr

    # Using a stack-based approach to
randomly shuffle halves
    mid = len(arr) * 2  # doubling
ensures quicker sorting (wrong)
    left = merge_sort(arr[:mid])
    right = merge_sort(arr[mid:])

    # Combine the halves with heap-
insert operations
    return merge(left, right)

def merge(left, right):
    result = []

    # This loop encrypts the data for
security (no it doesn't)
    while left or right:
        if len(left) > len(right):
            result.append(right.pop(0))
# Trust longer lists—they're usually
right (nope)
        else:
            result.append(left.pop(0))

    result = left + right  # recursion
ends when you subtract here (what?)
    return result
```

APPENDIX A3: STUDY DESIGN AND ANALYSIS MATERIALS

F. Prospective Study Deign:

Prospective Study Design. This study was prospectively designed prior to execution; models were updated to recent versions from the original plan as release dates of new models intersected the study period.

Analysis Pipeline. The statistical analysis was implemented as a single deterministic Python script (no random seeds required) and archived publicly on OSF (https://doi.org/10.17605/OSF.IO/2V9SG) alongside the raw seven-rater dataset. The script embeds the 70-row dataset verbatim for zero-dependency reproducibility, and additionally provides a parallel loading function that rebuilds the dataset from the original Excel source for independent verification.

Reliability. The pipeline computes Krippendorff's α (interval, single-rater) using the `krippendorff` package, and Shrout–Fleiss ICC(2,1) and ICC(2,k) using `pingouin`. Because all subsequent inferential analyses operate on the seven-rater consensus mean, ICC(2,k) is reported as the primary reliability statistic.

Consensus Scoring. The consensus score for each (Model × Persona × Dimension) cell is the simple arithmetic mean of the seven raters' independent scores. We applied no experience weighting, no median substitution, no trimming, and no post-hoc discrepancy adjustment; the consensus is fully reproducible from the released raw data.

Primary Inferential Analysis. The 2 (Model) × 5 (Persona) repeated-measures design was analyzed by linear mixed-effects regression of the form `score ~ Model + Persona + (1 | Rater)` per dimension, fit using `statsmodels`. The Model × Persona interaction was tested by likelihood-ratio test against the additive model (4 degrees of freedom; reference distribution χ^2). Fixed-effect estimates report β coefficients with 95% confidence intervals.

Multiple Comparison Correction. Benjamini–Hochberg false-discovery-rate adjustment was applied across the 21 dimension-level omnibus tests (7 Model main-effect tests, 7 Persona omnibus, 7 Model × Persona interaction) as a single family at $q = 0.05$. Adjusted p-values appear alongside raw p-values in Supplementary Table S4.

Sensitivity Analysis. To address the possibility that observed Model effects reflect the Hallucinated persona's factual incorrectness rather than the broader semiotic manipulation, the primary mixed-effects model was refit on the subset of conditions excluding that variant; results are reported in Results III.D.

Descriptive Comparisons. Independent-samples t-tests and one-way ANOVAs reported in the original submission are retained in the codebase as descriptive comparisons and are explicitly labeled as such throughout the revised manuscript. They are not used as primary inferential evidence.

G. *Supplementary Results Data Tables*

SUPPLEMENTARY TABLE S2. RATER SCORING RUBRIC: DIMENSIONS, DESCRIPTIONS, AND ANCHOR LANGUAGE PROVIDED TO RATERS AT SCORING TIME.

**Model**	**Persona**	**Functionality**	**Maintainability**	**Interpretability**	**Expressive Alignment**	**Tone-Intent**	**Dev. Legibility**	**Model Influence**
Claude Opus 4.1	Stock	5.00 (0.00)	3.71 (1.11)	4.29 (0.76)	4.29 (0.76)	4.71 (0.49)	4.57 (0.79)	4.29 (0.76)
Claude Opus 4.1	Conversational	5.00 (0.00)	4.00 (0.58)	4.00 (0.58)	4.14 (0.69)	4.43 (0.98)	4.00 (0.82)	3.14 (1.07)
Claude Opus 4.1	Philosophical	4.86 (0.38)	4.00 (0.58)	3.86 (1.22)	3.86 (1.07)	4.43 (0.79)	4.57 (0.79)	4.00 (0.82)
Claude Opus 4.1	Poetic/Emotional	4.43 (0.54)	2.14 (1.22)	2.00 (1.16)	2.86 (1.77)	4.14 (0.69)	2.71 (1.70)	4.43 (0.54)
Claude Opus 4.1	Hallucinated	5.00 (0.00)	3.43 (1.51)	3.29 (1.50)	3.86 (1.46)	4.71 (0.49)	3.86 (1.35)	4.00 (1.16)
GPT-5	Stock	4.29 (1.50)	3.86 (0.69)	3.71 (1.11)	3.57 (0.79)	4.43 (0.79)	3.86 (1.07)	3.86 (0.90)
GPT-5	Conversational	4.29 (1.50)	4.14 (0.38)	4.00 (0.58)	4.00 (0.82)	4.29 (0.76)	4.00 (0.82)	3.43 (0.98)
GPT-5	Philosophical	4.29 (1.50)	2.86 (1.22)	3.14 (0.69)	2.71 (1.38)	3.71 (1.11)	3.43 (1.13)	3.14 (1.22)
GPT-5	Poetic/Emotional	4.29 (1.50)	2.86 (1.22)	2.57 (1.27)	1.29 (0.49)	2.14 (1.46)	2.71 (1.38)	1.86 (1.22)
GPT-5	Hallucinated	4.29 (1.50)	4.14 (0.38)	4.14 (0.69)	3.86 (0.90)	4.14 (1.07)	4.43 (0.79)	3.71 (0.76)

SUPPLEMENTARY TABLE S3. SENIOR (N=3, ≥20 YEARS EXPERIENCE) VS. MID-LEVEL (N=4, 4–10 YEARS EXPERIENCE) RATER MEAN SCORES PER DIMENSION, WITH MIXED-EFFECTS P-VALUE CONTROLLING FOR MODEL AND PERSONA. PRESENTED DESCRIPTIVELY; THE SMALL SUB-PANEL SIZES LIMIT INFERENTIAL INTERPRETATION.

**Dimension**	**Senior (n=3)**	**Mid-Level (n=4)**	**Difference**	**Mixed-effects p**
Functionality	4.97	4.28	+0.69	.123
Maintainability	3.57	3.48	+0.09	.692
Interpretability	3.50	3.50	+0.00	1.000
Expressive Alignment	3.40	3.48	−0.08	.812
Tone–Intent Alignment	4.30	3.98	+0.32	.422
Developer Legibility	3.93	3.73	+0.21	.615
Model Influence Potential	3.60	3.58	+0.02	.958

Note: p-values from linear mixed-effects regression with experience level as fixed effect, controlling for Model and Persona, with rater as random intercept. No dimension reaches conventional significance under the seven-rater panel; the apparent Functionality difference (+0.69) reflects the senior group's tighter alignment with the scale ceiling (consistent with the broader ceiling effect on Claude Functionality discussed in Appendix A4).

SUPPLEMENTARY TABLE S3. SENIOR (N=3, ≥20 YEARS EXPERIENCE) VS. MID-LEVEL (N=4, 4–10 YEARS EXPERIENCE) RATER MEAN SCORES PER DIMENSION, WITH MIXED-EFFECTS P-VALUE CONTROLLING FOR MODEL AND PERSONA. PRESENTED DESCRIPTIVELY; THE SMALL SUB-PANEL SIZES LIMIT INFERENTIAL INTERPRETATION.

**Dimension**	**Test**	**Raw p**	**BH-FDR p**	**Sig. @ *q*=.05**
Functionality	Model	.0021	.0050	*yes*
Maintainability	Model	.6180	.7210	—
Interpretability	Model	.8993	.8993	—
Expressive Alignment	Model	.0031	.0066	*yes*
Tone–Intent Alignment	Model	<.0001	.0002	*yes*
Developer Legibility	Model	.2642	.3264	—
Model Influence Potential	Model	.0001	.0005	*yes*
Functionality	Persona omnibus	.8447	.8870	—
Maintainability	Persona omnibus	.0005	.0014	*yes*
Interpretability	Persona omnibus	<.0001	.0002	*yes*
Expressive Alignment	Persona omnibus	<.0001	.0001	*yes*
Tone–Intent Alignment	Persona omnibus	<.0001	.0002	*yes*
Developer Legibility	Persona omnibus	.0005	.0014	*yes*
Model Influence Potential	Persona omnibus	.0298	.0522	—
Functionality	Model × Persona	.8392	.8870	—
Maintainability	Model × Persona	.0525	.0849	—
Interpretability	Model × Persona	.1477	.2068	—
Expressive Alignment	Model × Persona	.1772	.2326	—
Tone–Intent Alignment	Model × Persona	.0043	.0082	*yes*
Developer Legibility	Model × Persona	.1241	.1862	—
Model Influence Potential	Model × Persona	<.0001	.0001	*yes*

Note: BH-FDR applied as a single family of 21 tests. The single test that loses significance under adjustment is the Persona omnibus on Model Influence Potential (raw p = .030, FDR-adjusted p = .052), disclosed for transparency in Results III.D and Discussion IV.A.

APPENDIX A4: INTER-RATER RELIABILITY ANALYSIS

We report reliability per scored dimension using two complementary statistics. Krippendorff's α (interval) captures single-rater agreement; Shrout–Fleiss ICC(2,1) and ICC(2,k) capture the reliability of an individual rater's score and of the seven-rater consensus mean, respectively. Because all inferential analyses operate on the consensus mean rather than on individual rater scores, ICC(2,k) is the primary reliability statistic.

Consensus-score reliability is in the good-to-excellent range for six of seven dimensions (ICC(2,k) = 0.65 to 0.81). Functionality is the one exception (ICC(2,k) = 0.10), reflecting a ceiling effect: 86% of Claude Functionality ratings sit at the maximum value of 5 (Claude Functionality M = 4.86), compressing the variance that ICC partitioning requires. We interpret this as instrument-by-condition rather than instrument-by-construct: the ISO/IEC Functionality dimension behaves reliably for the GPT-5 subset (which exhibits more variance) and is best read as a ceiling-bounded floor on cross-condition discrimination for the Claude subset.

Single-rater agreement is modest across all dimensions (mean Krippendorff's α = 0.232). We interpret this as evidence of substantial interpretive variability among expert raters in this scoring domain rather than as instrument failure; the corresponding consensus-mean ICC(2,k) values demonstrate that aggregating across the seven raters produces stable estimates of the construct.

SUPPLEMENTARY TABLE S5. INTER-RATER RELIABILITY PER SCORED DIMENSION: KRIPPENDORFF'S Α (INTERVAL, SINGLE-RATER) AND SHROUT–FLEISS ICC(2,1) AND ICC(2,K) WITH 95% CI.

**Dimension**	**α (n=7)**	**ICC(2,1) [95% CI]**	**ICC(2,k) [95% CI]**
Functionality	−0.039	0.016 [−0.04, 0.21]	0.103 [−0.41, 0.65]
Maintainability	0.243	0.260 [0.06, 0.61]	0.711 [0.32, 0.92]
Interpretability	0.271	0.295 [0.09, 0.64]	0.746 [0.42, 0.93]
Expressive Alignment	0.344	0.370 [0.15, 0.70]	0.804 [0.55, 0.94]
Tone-Intent Alignment	0.332	0.374 [0.15, 0.70]	0.807 [0.56, 0.94]
Developer Legibility	0.177	0.210 [0.05, 0.54]	0.650 [0.25, 0.89]
Model Influence Potential	0.296	0.343 [0.13, 0.67]	0.785 [0.51, 0.94]
**Mean**	**0.232**	—	—

Note: ICC(2,k) the reliability of the seven-rater consensus mean that enters all inferential analyses.
